# A recipe for postfledging survival in great tits *Parus major*: be large and be early (but not too much)

**DOI:** 10.1002/ece3.2192

**Published:** 2016-06-08

**Authors:** Samuel Rodríguez, Arie J. van Noordwijk, Elena Álvarez, Emilio Barba

**Affiliations:** ^1^ Department of Terrestrial Vertebrates‘Cavanilles’ Institute of Biodiversity and Evolutionary BiologyUniversity of ValenciaValenciaSpain; ^2^ Department of Animal EcologyNetherlands Institute of EcologyWageningenThe Netherlands; ^3^ Present address: Department of Evolutionary EcologyNational Museum of Natural Sciences, CSICJosé Gutiérrez Abascal 228006MadridSpain

**Keywords:** Breeding success, Cormack–Jolly–Seber models, fledging condition, hyperthermia, long‐term study

## Abstract

Survival of juveniles during the postfledging period can be markedly low, which may have major consequences on avian population dynamics. Knowing which factors operating during the nesting phase affect postfledging survival is crucial to understand avian breeding strategies. We aimed to obtain a robust set of predictors of postfledging local survival using the great tit (*Parus major*) as a model species. We used mark–recapture models to analyze the effect of hatching date, temperatures experienced during the nestling period, fledging size and body mass on first‐year postfledging survival probability of great tit juveniles. We used data from 5192 nestlings of first clutches ringed between 1993 and 2010. Mean first‐year postfledging survival probability was 15.2%, and it was lower for smaller individuals, as well as for those born in either very early or late broods. Our results stress the importance of choosing an optimum hatching period, and raising large chicks to increase first‐year local survival probability in the studied population.

## Introduction

Variation in juvenile survival has profound effects on avian population dynamics (Arcese et al. 1992; Robinson et al. 2004; Finkelstein et al. 2010). First‐year mortality after leaving the nest can be particularly high (Perrins 1979, 1980; Magrath 1991; Naef‐Daenzer et al. 2001), which may have major consequences for the proportion of recruits into the breeding population (Starck and Ricklefs 1998). In spite of its importance, the postfledging period has remained one of the least studied components of avian demographics due to logistic difficulties in monitoring individuals after leaving the nest (Drent 1984; Hannon and Martin 2006; Vitz and Rodewald 2011). Consequently, many studies have often relied on prefledging characteristics to predict the survival of offspring, or have used local return rates to estimate survival (e.g., Ashcroft 1979; DiCostanzo 1980; Nisbet et al. 1984). In this sense, the development of capture–recapture models and their application to ringing data obtained from long‐term studied populations have improved the reliability of the survival estimates, allowing the consideration of potential factors affecting postfledging survival (Lebreton et al. 1992; Skalski et al. 1993; White and Burnham 1999).

A common pattern found in several studies with passerines is a selection for early breeding (e.g., Naef‐Daenzer et al. 2001; Vitz and Rodewald 2011) and for a good condition at fledging, expressed through measures of fledging mass (e.g., Perrins 1965; Both et al. 1999; Monrós et al. 2002a) or skeletal body size (Brown and Brown 1998). Offspring fledging earlier in the season may benefit from milder environmental conditions, higher food availability (Krementz et al. 1989; Spear and Nur 1994; Naef‐Daenzer et al. 2001), reduced intraspecific competition for resources (Kluyver 1971; Matthysen 1990; Verhulst et al. 1995), as well as lower predation rates (Newton 1978; Naef‐Daenzer et al. 1999; Naef‐Daenzer et al. 2001) and parasitism (Burtt et al. 1991; Merino and Potti 1995; Verhulst and Nilsson 2008). There may also be differences in parental quality between early and late breeders, so that early chicks may receive a greater investment from their higher quality parents (Forslund and Pärt 1995; Hipfner 1997).

Deviations from the general pattern relating early breeding to high postfledging survival have been observed in different populations. Anders et al. (1997) did not find evidence of a seasonal change in juvenile survival of wood thrushes (*Hylocichla mustelina*, Gmelin 1789), whereas survival of juvenile brown thornbills (*Acannthiza pusilla*, White 1790) and lark buntings (*Calamospiza melanocorys*, Stejneger 1885) increased as the season progressed (Green 2001; Yackel Adams et al. 2006). Additionally, at least one study showed that very early blue tit (*Cyanistes caeruleus*, Linæus 1758) hatchlings might experience a reduced postfledging survival (Norris 1993). In the case of great tit (*Parus major*, Linæus 1758) fledglings, Monrós et al. (2002a) found that, depending on the year, either early, late, or mid‐season nestlings had more postfledging survival probabilities, and that temperatures experienced in the nest were in part responsible of these different patterns (Greño et al. 2008).

It is generally accepted that condition at fledging affects postfledging survival. Larger juveniles may be better suited to escape from potential predators (De Laet 1985), and a larger body mass could be advantageous to endure periods of food limitation (Perrins 1965; Blem 1990; Perrins and McCleery 2001). This correlation between body size and condition at fledging and postfledging survival implies that factors affecting chick development during the prefledging period may carry over to subsequent biological phases and compromise future reproduction (van der Jeugd and Larsson 1998; review in Harrison et al. 2011). In this sense, temperatures experienced during the nesting period could affect fledgling condition through their effect on chick growth and immunocompetence (Geraert et al. 1996; Dawson et al. 2005). Newly hatched altricial nestlings might experience higher vulnerability to adverse cold nest microclimates due to their inability to regulate metabolic heat production (Shilov 1973; O'Connor 1984; Rodríguez and Barba 2016), whereas high temperatures could affect grown nestlings of large broods, if they are unable to dissipate heat generated in excess (Mertens 1969; van Balen and Cavé 1970). Hyperthermia could be a serious issue in habitats such as those of the Mediterranean region, where maximum temperatures experienced during the breeding season may frequently exceed 30 °C, being thus liable to surpass the thermal tolerance of birds (Blondel et al. 1987; Belda et al. 1995; Greño et al. 2008). Previous manipulative studies in a Mediterranean great tit population have shown that exposition of nestlings to adverse high temperatures during development may not increase mortality in the nest, but rather lead to reduced mass at fledging, which could eventually lower first‐year survival probability (S. Rodríguez & E. Barba, unpubl. data).

Most analyses of juvenile postfledging survival are based on relatively short‐term data, which entails the risk of failing to account for all the variability in local survival trends, or ignoring certain factors affecting overall juvenile survival in favor of others that may only be relevant during specific years. Long‐time series are therefore necessary to clarify the main factors affecting first‐year postfledging survival of juveniles across different years, leading to a robust set of predictors of postfledging survival in a particular population.

Our aim here was to determine the effects of hatching date, temperatures experienced during nestling development, and fledgling mass and size on first‐year postfledging survival in a Mediterranean great tit population, using capture–recapture data from 21 years (1993–2013). Based on previous studies, we predicted that (1) both relatively high and relatively low ambient temperatures experienced during vulnerable periods of nestling development will have negative effects on juvenile survival, irrespective of dates; (2) the effect of dates per se will depend on the year (i.e., there will be years where early, late or mid‐season hatchlings will have better survival prospects); and (3) large and/or heavier fledglings would have more postfledging survival probabilities.

## Materials and Methods

We used data collected during a long‐term study of a great tit population breeding in nest boxes within an extensive orange monoculture in Sagunto (Valencia), Eastern Spain (39°42′N, 0°15′W, 30 m a.s.l.). Wooden nest boxes were placed each year for the birds to breed, and were visited with the periodicity necessary (at least weekly, and daily at some stages) to record basic breeding parameters (Greño et al. 2008; Álvarez and Barba 2014). Relevant to this work were exact hatching dates, obtained for all nests through daily visits around the expected date of hatching (day 0), and fledgling mass (digital balance, 0.01 g accuracy) and tarsus length (digital calliper, 0.01 mm accuracy), obtained from 15‐day‐old nestlings. Nestlings were ringed with individually numbered metal rings at this date. For survival analyses (see below), we used data from 5192 nestlings of 876 first clutches fledged between 1993 and 2010. Between 1994 and 2013, 508 of these individuals were recaptured as adult breeders. Of these, 332 (65.4%) were recaptured for the first time in the first year after fledging. The total number of captures and recaptures, considering one capture event per breeding season, was 5995 (4684 birds were ringed and never recaptured, 318 individuals recaptured only once, 119 twice, 48 three times, 14 four times, 7 five times, and 2 six times).

For each nest, we calculated average minimum ambient temperatures from hatching until nestlings were 5 days old, as well as average maximum ambient temperatures from day 10 to day 15. We considered these to be time periods of higher nestling vulnerability to low and high temperatures, respectively (Mertens 1969; Shilov 1973). Temperature data were collected from the meteorological station “Sagunto Pontazgo” close to the study area.

### Survival analyses

#### The general Cormack–Jolly–Seber modeling process

Previous studies with great tit fledglings have shown that the postfledging survival probabilities of juveniles of the same nest were independent from each other (Naef‐Daenzer et al. 2001). Moreover, results obtained in our population further support this finding (Monrós et al. 2002a). We thus considered individuals as independent units for analytical purposes. We used live recaptures models within the program MARK (White and Burnham 1999) to analyze postfledgling survival data. Our first step in the modeling process was to obtain a reference Cormack–Jolly–Seber (CJS) model (Lebreton et al. 1992), incorporating time dependency on local survival and recapture probabilities. Once we had this reference starting model, in a second step, we incorporated the effect of individual covariates, as described in White and Burnham (1999). To ensure that the numerical optimization algorithm finds the correct parameter estimates, the values of individual covariates were standardized using the option “Standardized Individual Covariates” from MARK. Model selection was based on Akaike's information criterion adjusted for sample size (AIC_c_, Burnham and Anderson 2002). The model with the lowest AIC_c_ represents the best balance between loss of precision (due to over fitting) and bias of the estimates (due to under fitting; Burnham and Anderson 2002). As general model selection criterion for analyses on postfledging survival probability (i.e., tests for time dependence and effect of individual covariates, as described below), models with a difference in AIC_c_ of less than two units were considered to be similarly supported by the data. Although models ranked within two and seven units from the best‐fitting model may also have some support (Burnham and Anderson 2011), likelihood ratio tests (LRTs) comparing these models with nested models from the selected subset were consistent with our more restrictive criterion, as none of the lower‐ranked models contributed significantly to variation in the data. Among the models within two AIC_c_ units, we chose the one with the fewest parameters as the best one explaining the data (Burnham and Anderson 2002), and checked whether this decision originated a significant loss of fit using a LRT.

#### Testing for time dependence of local survival and recapture probabilities

We created a series of general models incorporating time‐dependent effects on survival and recapture probabilities. These models were constructed using the sin link function. Our initial model considered time dependence in both survival and recapture probabilities, Ф(*t*)*p*(*t*). The validity of this simple model to the data was assessed by goodness‐of‐fit tests of program RELEASE in MARK (Burnham et al. 1987). The model fitted the data poorly (TEST 2 + TEST 3, *χ* ^2^ = 448.84, df = 61, *P* < 0.001). Results of TEST 3 (*χ* ^2^ = 394.67, df = 17, *P* < 0.001) suggested possible age effects on survival probabilities. Given that an individual's first‐year survival is likely to differ from that of older birds, we built a model incorporating two age classes: a_1_ (first‐year survival) and a_2_ (adult survival). In this model, we considered first‐year postfledging survival to be time dependent, and adult survival to be constant, as the analysis of time effects on older age classes was outside the objectives of our study. Recapture probability was considered to be time dependent. The goodness‐of‐fit of this new model, Ф(a_1_t, a_2_)*p*(*t*), was tested using the parametric bootstrap approach implemented in MARK. The parameter estimates from the model were used to simulate data according to the assumptions of CJS models (i.e., no over dispersion is included, animals are totally independent, and no violations of model assumptions are included). This process was repeated 1000 times, and the deviance of each model was calculated to determine whether the deviance of the observed model exceeded that of simulated data. The probability of obtaining by chance a deviance value as large as or larger than the one observed was given by the ratio between the number of simulations with deviance larger than the one observed in our general model divided by the total number of simulations. We established a significance level *P* < 0.05 for rejecting the null hypothesis. The bootstrap goodness‐of‐fit test indicated that the model had a good fit (*P* = 0.74), so we selected it as our reference model, and compared it with simpler nested models, using AIC_c_ values for model selection.

#### Testing for the effect of covariates on postfledging local survival

We used an information‐theoretic approach (Burnham and Anderson 2002) to examine first‐year postfledging survival of great tits in relation to hatching date, mass, tarsus length, average minimum temperatures during days 0 to 5 of age, and average maximum temperatures during days 10 to 15 of age. Starting with the best‐fitting time‐dependent general model from the previous step, we created a set of a priori‐hypothesized models where first‐year postfledging survival was dependent on different combinations of these individual covariates, never including in a single model both temperature variables. We also evaluated possible quadratic effects. Models including covariates were built with the logit link function to constrain the survival probability to a value between zero and one. To limit the set of models analyzed and simplify interpretation, we only included interaction terms in case we considered them relevant, in view of the results and/or their biological meaning. We created a total of 23 models and ranked them according to their AIC_c_ values. When estimating the effect of an individual covariate on a model, we assumed that when the 95% confidence interval of its *β*‐parameter (as provided in program MARK output for each of the covariates included in a model, see Franklin 2001) included zero, it meant weak or no effect of that covariate on first‐year postfledging survival (e.g., Traylor et al. 2004).

## Results

### Survival and recapture probabilities

The best‐fitting general model had constant first‐year and adult survival probabilities (Table 1, Model 1 vs. Model 3), and time variation in recapture probabilities (Table 1, Model 1 vs. Model 2). Based on this model, first‐year postfledging local survival probability (±SE) was 15.2 ± 0.8%, whereas adult survival probability was 56.1 ± 1.6%. Estimated recapture probabilities ranged between 28.4% in 2004 and 72.8% in 2003, with a mean of 44.5 ± 11.8%.

**Table 1:** Model selection for time‐dependent effects on recapture and first‐year postfledging survival probabilities of great tits breeding in eastern Spain. For each model, the values of Akaike's information criterion (AIC_c_), difference of AIC_c_ values in relation to the best‐fitting model (∆AIC), AIC weights, number of estimable parameters (*N*_p_), and deviance (DEV) are shown. Model notation is as follows: Ф, survival probability; *p*, recapture probability; *t*, time dependence (year); a_1_, first‐year survival probability of fledglings; a_2+_, survival probability of adults. Selected model in bold

Models	AIC_c_	∆AIC	AIC weight	*N*_p_	DEV
Modeling recapture probability					
1. $\Phi_{\left(a_{1},a_{2+}\right)}p_{\left(t\right)}$	5763.56	0.00	0.97873	22	523.31
2. $\Phi_{\left(a_{1},a_{2+}\right)}p_{\mathrm{constant}}$	5791.36	27.80	0.00000	3	589.27
Modeling survival probability					
**1.** $\Phi_{(a_{1},a_{2+})p_{\left(t\right)}}$	**5763.56**	**0.00**	**0.97873**	**22**	**523.31**
3. $\Phi_{\left(a_{1t},a_{2+}\right)}p_{\left(t\right)}$	5771.22	7.66	0.02127	40	494.58

### Covariates affecting postfledging survival

To determine which of the studied covariates had a significant effect on first‐year survival probability, we created separate models including the effect of each individual covariate on first‐year postfledging survival (Table 2, Models 9, 15, 17, 20 and 21), and compared them with the reference general model from the previous step (Table 2, Model 18). Models including an effect of tarsus length, hatching date, and average minimum temperature during days 0–5 had a better fit to the data than the reference model, so we considered these covariates relevant. When compared individually, models with tarsus length fitted the data better than models with either hatching date or minimum temperature (Table 2, Model 9 vs. Model 15, Model 9 vs. Model 17). Contrasting the effect of temperature vs. that of date, a model including only the effect of hatching date on first‐year postfledging survival probability had a significantly lower AIC (Model 15 vs. Model 17, ∆AIC = 4), and received seven times more support than a model including *t*_min_. The fit of these two latter models improved significantly with the introduction of quadratic effects (hatching date: Model 14 vs. Model 15, ∆AIC = 3.42; minimum temperature: Model 12 vs. Model 17, ∆AIC = 8.94). On the other hand, models including mass and average maximum temperature during days 10–15 received higher AIC_c_ scores than the reference model, and therefore, we considered these covariates to have no significant direct effect on first‐year postfledging survival probability. The inclusion of quadratic effects improved nonsignificantly the fit of the model in the case of maximum temperature (Model 19 vs. Model 21, ∆AIC = 0.89), and did not improve model fit in the case of mass (Model 20 vs. Model 22, ∆AIC = 1.93). Of the two biometrical covariates, a model including the effect of tarsus length on first‐year postfledging survival explained data 462 times better than a model including the effect of mass.

**Table 2:** Model selection for effects of covariates on first‐year postfledging survival probabilities of great tits breeding in eastern Spain. For each model, the values of Akaike's Information Criterion (AIC_c_), difference of AIC_c_ values in relation to the best‐fitting model (∆AIC), AIC weights, number of estimable parameters (*N*_p_), and deviance (DEV) are shown. Model notation is as follows: Ф, survival probability; *p*, recapture probability; *t*, time dependence (year); a_1_, first‐year survival probability of fledglings; a_2+_, survival probability of adults; +, additive factors; *, interaction; *t*_max_, average maximum temperatures during days 10–15 of age of nestlings; *t*_min_, average minimum temperatures during days 0–5 of age of nestlings; hd, hatching date; w, mass at fledging; tar, tarsus length at fledging. Covariates starting with sq mean squared effect of a covariate. Selected model in bold

Models	AIC_c_	∆AIC	AIC weight	*N*_p_	DEV
1. $\Phi_{\left(a_{1}\left(\mathrm{tar}+\mathrm{hd}+\mathrm{sqhd}+t_{\min}+\mathrm{sq}t_{\min}\right),a_{2+}\right)}p_{\left(t\right)}$	5746.71	0.00	0.31746	27	5692.45
2. $\Phi_{(a_{1}\left(\mathrm{tar}+\mathrm{hd}+\mathrm{sqhd}\right),a_{2+})}p_{\left(t\right)}$	**5747.32**	**0.61**	**0.23353**	**25**	**5697.10**
3. $\Phi_{\left(a_{1}\left(\mathrm{tar}+\mathrm{hd}+\mathrm{sqhd}+t_{\min}\right),a_{2+}\right)}p_{\left(t\right)}$	5748.42	1.71	0.13477	26	5696.18
4. $\Phi_{\left(a_{1}\left(\mathrm{tar}+\mathrm{hd}\right),a_{2+}\right)}p_{\left(t\right)}$	5749.54	2.84	0.07688	24	5701.34
5. $\Phi_{\left(a_{1}\left(\mathrm{hd}+\mathrm{sqhd}+t_{\min}+\mathrm{sq}t_{\min}\right),a_{2+}\right)}p_{\left(t\right)}$	5749.67	2.97	0.07207	26	5697.44
6. $\Phi_{\left(a_{1}\left(\mathrm{tar}+\mathrm{hd}+t_{\min}\right),a_{2+}\right)}p_{\left(t\right)}$	5750.48	3.77	0.04814	25	5700.26
7. $\Phi_{\left(a_{1}\left(\mathrm{tar}+t_{\min}+\mathrm{sq}t_{\min}\right),a_{2+}\right)}p_{\left(t\right)}$	5750.88	4.17	0.03940	25	5700.66
8. $\Phi_{\left(a_{1}\left(\mathrm{tar}*\mathrm{hd}\right),a_{2+}\right)}p_{\left(t\right)}$	5751.44	4.74	0.02975	25	5701.22
9. $\Phi_{\left(a_{1}\left(\mathrm{tar}\right),a_{2+}\right)}p_{\left(t\right)}$	5752.97	6.26	0.01387	23	5706.78
10. $\Phi_{\left(a_{1}\left(\mathrm{tar}+t_{\min}\right),a_{2+}\right)}p_{\left(t\right)}$	5753.60	6.90	0.01010	24	5705.40
11. $\Phi_{\left(a_{1}\left(\mathrm{tar}+\mathrm{sqtar}\right),a_{2+}\right)}p_{\left(t\right)}$	5753.84	7.13	0.00896	24	5705.64
12. $\Phi_{\left(a_{1}\left(t_{\min}+\mathrm{sq}t_{\min}\right),a_{2+}\right)}p_{\left(t\right)}$	5754.46	7.75	0.00658	24	5706.26
13. $\Phi_{\left(a_{1}\left(\mathrm{tar}*t_{\max}\right),a_{2+}\right)}p_{\left(t\right)}$	5755.41	8.70	0.00409	25	5705.19
14. $\Phi_{\left(a_{1}\left(\mathrm{hd}+\mathrm{sqhd}\right),a_{2+}\right)}p_{\left(t\right)}$	5755.98	9.27	0.00308	24	5707.77
15. $\Phi_{\left(a_{1}\left(\mathrm{hd}\right),a_{2+}\right)}p_{\left(t\right)}$	5759.40	12.69	0.00056	23	5713.21
16. $\Phi_{\left(a_{1}\left(\mathrm{hd}+t_{\min}\right),a_{2+}\right)}p_{\left(t\right)}$	5759.67	12.97	0.00049	24	5711.47
17. $\Phi_{\left(a_{1}\left(t_{\min}\right),a_{2+}\right)}p_{\left(t\right)}$	5763.40	16.69	0.00008	23	5717.21
18. $\Phi_{\left(a_{1},a_{2+}\right)}p_{\left(t\right)}$	5763.56	16.85	0.00007	22	5719.39
19. $\Phi_{\left(a_{1}\left(t_{\max}+\mathrm{sq}t_{\max}\right),a_{2+}\right)}p_{\left(t\right)}$	5764.43	17.73	0.00004	24	5716.23
20. $\Phi_{\left(a_{1}\left(w\right),a_{2+}\right)}p_{\left(t\right)}$	5765.08	18.38	0.00003	23	5718.90
21. $\Phi_{\left(a_{1}\left(t_{\max}\right),a_{2+}\right)}p_{\left(t\right)}$	5765.32	18.61	0.00003	23	5719.13
22. $\Phi_{\left(a_{1}\left(w+\mathrm{sq}w\right),a_{2+}\right)}p_{\left(t\right)}$	5767.01	20.30	0.00001	24	5718.81
23. $\Phi_{\left(a_{1}\left(w*t_{\max}\right),a_{2+}\right)}p_{\left(t\right)}$	5768.25	21.54	0.00001	25	5718.03

**Figure 1:**
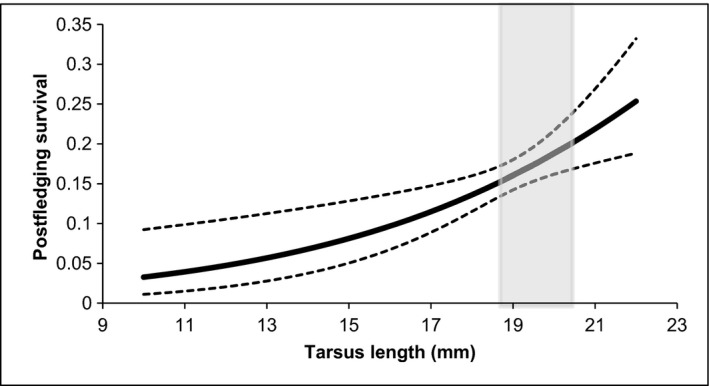
Effect of tarsus length on first‐year postfledging survival probability of great tits breeding in eastern Spain, as calculated by the program MARK model $\Phi_{\left(a_{1}\left(\mathrm{tar}+\mathrm{hd}+\mathrm{sqhd}\right),a_{2+}\right)}p_{\left(t\right)}$. Dotted lines represent the 95% CI. Shaded area includes approximately 80% of chicks.

**Figure 2:**
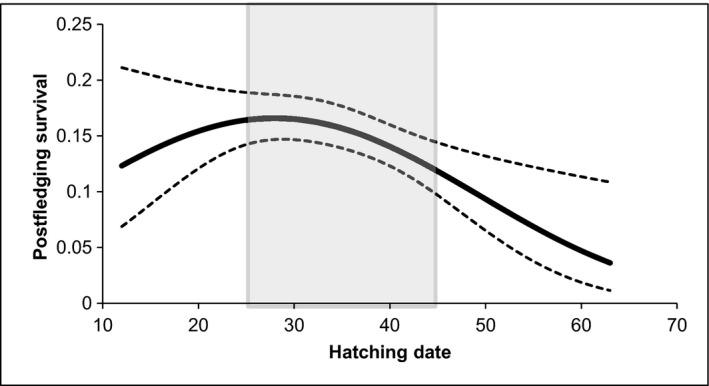
Effect of hatching date on first‐year postfledging survival probability of great tits breeding in eastern Spain, as calculated by the program MARK model $\Phi_{\left(a_{1}\left(\mathrm{tar}+\mathrm{hd}+\mathrm{sqhd}\right),a_{2+}\right)}p_{\left(t\right)}$. Dotted lines represent the 95% CI. Shaded area includes approximately 80% of chicks.

Our next step in fitting models was to consider different additive combinations of the relevant covariates, and testing whether the results improved by including quadratic effects. Our three best‐fitting models were similarly supported by the data, as their ∆AIC < 2 (Table 2, Models 1, 2 and 3). Together, their combined Akaike weight was 0.686. The three models incorporated tarsus length, hatching date, and hatching date squared and differed in the inclusion of minimum temperatures. The removal of *t*_min_ had no significant effect on the fit of the model, as judged by the LRT Test (Model 1 vs. Model 2: *χ* ^2^ = 4.650, df = 2, *P* = 0.0978; Model 3 vs. Model 2: *χ* ^2^ = 0.918, df = 1, *P* = 0.3380), and consequently the model with the fewer parameters (i.e., Model 2) was used to explain the effect of covariates on first‐year postfledging survival. In addition, we tested for a possible interaction between tarsus length and hatching date on first‐year survival, but the resulting model (i.e., Model 8) received no convincing support, as its ∆AIC was 4.74 and the 95% confidence interval of the *β*‐parameter of the interaction term included zero. Moreover, as the adverse effect of high temperatures on chick fitness may be aggravated during the late nestling stage depending on their size and overall ability to dissipate heat in excess (see van Balen and Cavé 1970), we also considered relevant to test for interactions between size (tarsus length or weight) and maximum temperatures. We found no convincing evidence to support these interactions, as the ∆AIC of the resulting models (Table 2, Models 13 and 23) was 8.70 and 21.54, respectively, and the 95% confidence interval of the *β*‐parameter of the interaction terms overlapped zero. According to the best‐ranked model, tarsus length and hatching date had a significant influence on first‐year survival probability, as their *β*‐terms did not overlap zero (Table 3). First‐year postfledging survival increased with nestling size (Fig. 1), and varied with hatching date following a nonlinear trend (Fig. 2). The effect of date on first‐year survival was such that hatching too early in the season, as well as hatching late, would have negative consequences on postfledging survival (Fig. 2). It is important to note that, regardless of the great dispersion in hatching dates in our study sample, the vast majority of chicks hatched during the “optimum” period leading to higher survival probability (i.e., April 21 to May 15), and that roughly <12% of the juveniles could be considered as being raised very early or late in the season. These marginal individuals also attained smaller sizes at fledging.

**Table 3:** *β*‐parameters (±SE) and 95% CI (in brackets) for the covariates of the best‐fitting models. Selected model in bold

Model	tarsus	hd	sqhd	*t*_min_	sq*t*_min_
**1.** $\Phi_{\left(a_{1}\left(\mathrm{tar}+\mathrm{hd}+\mathrm{sqhd}+t_{\min}+\mathrm{sq}t_{\min}\right),a_{2+}\right)}p_{\left(t\right)}$	0.13 ± 0.06 (0.01 to 0.24)	−0.12 ± 0.06 (−0.23 to −0.02)	−0.08 ± 0.04 (−0.159 to 0.002)	0.21 ± 0.10 (0.02 to 0.40)	0.07 ± 0.04 (−0.0005 to 0.1493)
**2.** $\Phi_{\left(a_{1}\left(\mathrm{tar}+\mathrm{hd}+\mathrm{sqhd}\right),a_{2+}\right)}p_{\left(t\right)}$	**0.17 ± 0.05 (0.07** to **0.28)**	−**0.14 ± 0.06 (**−**0.24** to −**0.03)**	−**0.08 ± 0.04 (**−**0.163** to −**0.002)**	–	–
**3.** $\Phi_{\left(a_{1}\left(\mathrm{tar}+\mathrm{hd}+\mathrm{sqhd}+t_{\min}\right),a_{2+}\right)}p_{\left(t\right)}$	0.17 ± 0.05 (0.06 to 0.27)	−0.13 ± 0.06 (−0.24 to −0.02)	−0.08 ± 0.04 (−0.1617 to −0.0003)	0.05 ± 0.05 (−0.05 to 0.15)	–

## Discussion

Our results suggest that hatching date and fledgling size (tarsus length) have a significant impact on first‐year postfledging survival probability. Smaller individuals, as well as those pertaining to either too‐early or late broods would have lower survival prospects. The effect of other potential covariates affecting first‐year survival, such as fledgling mass or temperatures experienced during the nestling stage, has not received convincing support. This way, of the two possible descriptors of body condition, fledgling size has proven to be a better predictor of first‐year postfledging survival than fledgling mass, and we have been unable to show the existence of carryover effects of either maximum or minimum ambient temperatures experienced during vulnerable periods of nestling development on first‐year survival probability.

### Effect of date on local first‐year survival probability

The effect of hatching date on first‐year local survival was nonlinear, suggesting there being an optimal range of breeding dates leading to a maximum first‐year postfledging survival probability, and that both positive and negative deviations from this range are not beneficial.

Birds have a limited period each year in which conditions for growth and reproduction are most suitable. In this sense, timing of breeding is essential, and individuals capable of adjusting their breeding schedule to match nestling development with the seasonal peak of prey availability will likely be able to raise larger fledglings of higher quality (van Noordwijk et al. 1995). Based on the results of this study (i.e., most of the chicks hatched during the optimum period), the majority of females in our great tit population were able to successfully track environmental change and raise their chicks when breeding conditions were finest.

The seasonal decline in breeding productivity is a common trend among avian populations (Perrins 1965; Nilsson and Smith 1988; Daan et al. 1989; Verhulst and Tinbergen 1991; Naef‐Daenzer et al. 2001). It is argued that juveniles from later broods suffer higher predation rates (Newton 1978; Naef‐Daenzer et al. 1999; Sim et al. 2012) and detrimental environmental conditions (Naef‐Daenzer et al. 2001; Öberg et al. 2014). The causal relationship between poor breeding performance and late breeding has also been supported in our study site, as delayed great tit pairs have been shown to produce fewer fledglings, of lower quality, that were less likely to be recruited into the local breeding population (Barba et al. 1995). The present study reveals that not only late breeding, but also very early breeding, entails lower first‐year postfledging survival probability. This finding would be in agreement with previous findings in blue tits (Norris 1993). Although this is a relatively old study, we did not find other ones demonstrating that breeding too early was disadvantageous. We believe that very early broods may be more likely exposed to sudden episodes of environmental instability, which are frequent in our study site at the beginning of the spring. These episodes, although of short duration, are characterized by strong temperature drops and intervals of heavy rain, and may pose a serious threat to developing chicks, thus endangering future survival prospects. The influence of hatching date on postfledging survival was also suggested by Monrós et al. (2002a), although its effect (either positive or negative) could vary from year to year, and no clear overall pattern emerged. The consideration of a longer dataset has helped to highlight the advantage of early fledging on first‐year survival, but also that juveniles hatching too early could be penalized as well.

On the other hand, Greño et al. (2008) took into account the potential effect of ambient temperatures experienced during the nestling stage on first‐year postfledging survival, and suggested the existence of both direct (i.e., increasing thermal stress) and indirect effects (i.e., through effects on food availability) of temperatures on first‐year survival probability. We have been unable to find evidence for date‐independent thermal effects, even after considering shorter periods of high nestling vulnerability to suboptimal temperatures. Maximum temperature was discarded in the first steps of model fitting and, although minimum temperature was a covariate included in two of the three best‐scored models (Table 2, Model 1 and Model 3), its exclusion did not lead to a significant loss of fit. Our results support that the effect of ambient temperatures during the nesting period on postfledging survival found in our study site is a consequence of their correlation with dates and more likely to be indirect, that is, a result of changing environmental conditions at fledging as the season progresses.

### Effect of fledgling size on local first‐year survival probability

Juvenile size at fledging had a positive effect on first‐year postfledging survival probability. Larger individuals may be less vulnerable to diseases, parasites and predators during their first months of life (Ragusa‐Netto 1996; van der Jeugd and Larsson 1998). They may also be favored during severe weather conditions, due to their greater capacity to retain heat and store fat (Brown and Brown 1998). Additionally, body size has been shown to be directly related to the establishment of dominance relationships between juveniles during the postfledging period, as larger fledglings tend to dominate over smaller ones (Garnett 1981). This superiority allows bigger fledglings to reach full independence in better physical condition than their weaker siblings (Kitowski 2005; Vergara and Fargallo 2008), thus improving long‐term survivorship (Arcese and Smith 1985; Desrochers et al. 1988; Piper and Wiley 1990). Moreover, the absence of evidence for an interaction between date and body size on postfledging survival probability suggests that large fledglings have higher first‐year survival than their smaller siblings with independence of the date they were born. In this sense, it is important to note that, late in the season, few chicks eventually develop large body sizes at fledging in our population (e.g., only 14% of fledglings hatched after May 15 have tarsi > 20 mm).

The relationship between size and postfledging survival has been documented in numerous studies, although it is common to express body size in terms of fledgling mass (Garnett 1981; Ragusa‐Netto 1996; Velando 2000). In our case, tarsus length proved to be much better at predicting first‐year postfledging survival than mass (it was the single most important variable affecting first‐year survival), probably because it is a more accurate indicator of overall chick size. In this sense, skeletal body size of juveniles at fledging is not likely to vary during their transition to adulthood; it is therefore a final measurement of juvenile size, whereas initial body mass differences between fledglings could be compensated during the postfledging period depending on food availability. Monrós et al. (2002b) showed that great tit fledgling mass may vary during the immediate days after leaving the nest (i.e., lighter than average chicks will tend to gain mass, whereas heavier than average birds will tend to lose it). Our results suggest that measures of skeletal body size should be provided when analyzing postfledging survival in relation to fledging characteristics, as they are more consistent estimators of individual body size at fledging.

In conclusion, we highlight the importance of hatching date and body size as determinants of first‐year survival in a Mediterranean great tit population. Large fledglings hatched between April 21 and May 15 have greater first‐year postfledging survival probabilities, most likely as a result of superior fitness and competitive skills, as well as more favorable environmental conditions at fledging.

## Conflict of Interest

None declared.
